# Safety of high-risk diabetic patients during Ramadan at a tertiary care hospital in Pakistan, practicing updated IDF DAR guidelines

**DOI:** 10.12669/pjms.40.5.8007

**Published:** 2024

**Authors:** Qaisar Farooq, Tahir Ghaffar, Suleman Elahi Malik, Aziz ul Hasan Aamir

**Affiliations:** 1 Qaisar Farooq, MBBS, FCPS. Endocrine Fellow, Department of Diabetes and Endocrinology, Hayatabad Medical Complex, Peshawar, Pakistan; 2 Tahir Ghaffar, MBBS, FCPS, MRCP. Department of Diabetes, Endocrinology and Metabolic Diseases, MTI Hayatabad Medical Complex, Peshawar, Pakistan; 3 Suleman Elahi Malik, MBBS, FCPS. Department of Endocrinology, MTI Khyber Teaching Hospital, Peshawar, Pakistan; 4 Azizul Hasan Aamir, MRCP, FRCP, FACE. Department of Diabetes, Endocrinology and Metabolic Diseases, MTI Hayatabad Medical Complex, Peshawar, Pakistan

**Keywords:** Diabetes Mellitus, Ramadan, High risk patients, Safety

## Abstract

**Objective:**

To determine trend of following medical advice and safety of high-risk diabetic patients during Ramadan practicing updated IDF DAR guidelines

**Methods:**

The cross-sectional study was conducted at Outpatient Department of Diabetes, Endocrinology and Metabolic Disorders Hayatabad Medical Complex Peshawar, Pakistan from April to June 2022, and comprised of high-risk diabetic patients (>6.0) based on updated IDF-DAR guidelines 2022 intending to fast. A questionnaire was designed to document patient risk factors score, type and duration of diabetes, HbA1c, comorbidities and complications developed during Ramadan. Data was analyzed using SPSS 20.

**Results:**

Among all 130 participants, 78(60%) followed medical advice and did not fast and 52(40%) patients fasted against medical advice during month of Ramadan. Out of 130 participants, 89.2% were having type-2 diabetes Mellitus, 55.4% were female and mean age of participants was 52+14.6.40%. In fasting group, 57.7% were in the age range of 16 to 50 years while in non-fasting group 69.2% participants were more than 50 years old (P-value 0.031). There were 80.8% female participants in fasting group versus 38.5% in non-fasting group (P-value 0.001). Hypoglycemia occurred in 58.3% patients in fasting group and 29.3% non-fasting group. (P-value 0.021). On the other hand, 27.8% patients in fasting group and 55.2% of non-fasting group had hyperglycemia (P-value 0.025).

**Conclusion:**

Despite advised against fasting in these high-risk patients as per IDF DAR guidelines, almost half of patients fasted considering fasting a religious obligation. Those who fasted had significant hypoglycemia despite adjustment of medications as in guidelines. There is need of more intensive education before fasting, especially in high-risk diabetic patients.

## INTRODUCTION

The prevalence of diabetes mellitus (DM) rising exponentially and it’s effecting every continent of the world with no exceptions. There are more than 400 million people with diabetes living across the world and this number will cross 700 million expectedly by 2045 with more than 50% increase, which is very high.^1^ The number of Muslims living in the world is approximately 1.9 billion, which is about one fourth of the world’s population.^2^ The majority Muslim population living in the Middle East (93%), Central Asia (83%), Southeast Asia (42%), South Asia (31%), Sub-Saharan Africa(31%) and all these regions are heavily impacted by diabetes mellitus.^3^ Pakistan is ranked at 3^rd^ position in DM prevalence after China and India having thirty three (33) million people with diabetes as per International Diabetes Federation (IDF) statistics.^4^

Fasting during Ramadan is one of the religious obligation each year where Muslims fast from dusk to dawn for a month. Fasting starts from morning meal at dawn called sahoor and ends at evening meal at dusk called iftar. It is one of the basic foundations of Islam being in the five pillars of Islam so there is intense desire to fast at all ages after puberty. There are some exemptions like for elderly, children and pregnant women who can compensate by donating money or food to the poor people.^3^ Muslims across the world try their best to fast in Ramadan. A study conducted in 39 countries enrolling 38,000 Muslims reported that 93% fasted during Ramadan revealing importance of the matter.^5^

Ramadan fasting affects several physiological functions of the body due to alteration of meal timings, sleep timings, energy and fluid balance especially in summer months with long fasting hours and hot weather especially in this part of the world as it rotates per lunar calendar.^3^,^6^ Fasting is specially challenging for patients with DM as risk of both hypoglycemia and hyperglycemia is increased and there are alterations in inflammatory markers as well.^7^During fasting hours, there is an increased risk of hypoglycemic episodes due to reduced oral intake. At iftar, there is a tendency to eat large portions with high carbohydrate intake, and this can increase the risk of hyperglycemic episodes. This altered homeostasis increases the risk of complications like dehydration, thromboembolism and diabetic ketoacidosis.^8^

This necessitated the urge for evidence-based guidelines to stratify people based on health status and treatment to decide about fasting. IDF-DAR (Diabetes and Ramadan) guidelines were first published in 2016 to provide essential roadmap to health care professionals to help patients with diabetes for fasting safely during Ramadan. The second edition of these guidelines were published in 2021which introduced scoring system for risk categorization. The basic concept is to involve people intending to fast six to eight weeks prior to Ramadan and investigate to stratify them into low, medium and high-risk categories. Those in low and medium risk category can fast after medications adjustment, dietary education, regular glucose record and to breakfast in case severe hypo and hyperglycemia. Those in high-risk category are strongly discouraged against fasting.^3^

Despite this, patients in high risk still desire to fast which put them at very high risk of complications. This study was designed to study this category of patients, who were in high-risk category based on IDF-DAR recent second edition risk score, and still decided to fast and followed throughout Ramadan to document frequency and type of complications as well as the trend to follow medical advice in our local population.

## METHODS

This is a cross sectional study was conducted from April to June 2022 in the Outpatient Department of Diabetes, Endocrinology and Metabolic Diseases, MTI Hayatabad Medical Complex Peshawar. Duration of study was 10 weeks including month of Ramadan. Data was collected four weeks before Ramadan. Total of 130 patients fulfilling inclusion criteria that is diabetic patients in high-risk category (>6.0) based on IDF-DAR guidelines and patients with type-2 DM, type I DM, gestational DM were included in study.

### Ethical Approval:

It was obtained from the Ethics committee of Hayatabad Medical Complex, Peshawar with reference no 650/HEC/B&PSC/2022 on 5^th^ April 2022.

Similarly, Diabetic patients with low or moderate risk (<6.0) based on IDF-DAR risk calculator and critically ill patients currently having stroke, hyperosmolar hyper glycemic state, diabetic ketoacidosis, acute kidney injury, sepsis, amputation was excluded. A written informed consent was obtained from all the patients enrolled in our study. The study population was strictly comprising of patients satisfying the inclusion and exclusion criteria. A detailed history was taken and physical examination was performed, and all the relevant information was recorded in a well-designed questionnaire. The patient record was assessed for glycemic status and any previous workup done regarding diabetic complications. Baseline investigation including Renal Function test, eGFR, HbA1C was sent. Hypoglycemia was blood glucose level <70mg/dl and hyperglycemia were blood glucose level >300mg/dl.^9^ Data was stored and analyzed by the statistical program SPSS version 20.0.

## RESULTS

The baseline demographics and clinical characteristics of the studied population are shown in Table-I and Table-II. In total 138 participants of study, eight patients were lost to follow-up. Out of the 130 patients, 72(55.4%) were females. Overall mean age was 52±14.96. T2DM was predominant 116(89.2%); All patients were on insulin including basal bolus (40%), premix insulin (53.8%), and basal only (6.2%). About 50.8 % of patients were also taking oral anti diabetics. Mean insulin dose was 39.17+14.68 units. Based on HbA1c, 26 (20%) patients had controlled DM (<9) while 104(80%) had uncontrolled DM (>9). Twelve patients were pregnant and 52 (40%) patients had either stable or unstable macro vascular disease.

**Table-I:** Clinical and biochemical characteristics of study participants.

Parameter	Fasting	Non-Fasting	Total
No of patients(n)	52(40 %)	78(60 %)	130(100)
Age	44.38±15.39	57.08±11.966	52.0±14.69
HbA1c	10.2954±1.568	11.12±2.198	10.79±2.00
Insulin Dose	36.85±15.932	40.72±13.793	39.17±14.68
Risk score	10.6154±3.525	11.2436±2.858	10.99±3.13

**Table-II:** Demographic and clinical features of the studied population.

Parameters	Fasting n (%)	Non-Fasting n (%)	Total n (%)	P-value
***Age (years)***				0.031
16-50	30(57.7)	24(30.8)	54(41.5)	
>50	22(42.3)	54(69.2)	76(58.5)	
***Duration of DM (years)***				0.188
1-10	30(57.7)	32(41.0)	62(47.7)	
>10	22(42.3)	46(59.0)	68(52.3)	
***Type of DM***				0.072
Type I DM	10(71.4)	4(28.6)	14(10.8)	
Type-2 DM	42(28.6)	74(63.8)	116(89.2)	
***Gender***				0.001
Male	10(19.2)	48(61.5)	58(44.6)	
Female	42(80.8)	30(38.5)	72(55.4)	
***MVD***				0.836
Yes	20(38.5)	32(41.0)	52(40)	
No	32(61.5)	46(59.0)	78(60)	
***Control of DM (HbA1c)***				0.255
<9	14(26.9)	12(15.4)	26(20.0)	
>9	38(73.1)	66(84.6)	104(80.0)	
***Type of Insulin***				0.059
Basal bolus	30(57.7)	22(42.3)	52(40.0)	
Premix	20(28.6)	50(71.4)	70(53.8)	
Basal Only	2(25.0)	6(75.0)	8(6.2)	
***Follow-up***				
***Hypoglycemia***				0.021
Yes	28(58.3)	24(29.3)	52(40.0)	
No	20(41.7)	58(70.7)	78(60.0)	
***Hypoglycemia assistance***				0.159
Yes	22(52.4)	20(47.6)	42(36.0)	
No	30(34.1)	58(65.9)	88(64.0)	
***Hyperglycemia***				0.025
Yes	20(27.8)	32(55.2)	52(55.4)	
No	52(42.3)	26(44.8)	78(44.6)	

Among all participants, 78 (60%) followed medical advice and did not fast and 52(40%) patients fasted during month of Ramadan. Mean number of fast days were 24.88±9.23. Mean difference in the fasting and non-fasting groups were, age 44.38±15.32 and 57.08±11.96, HbA1c 10.29±1.56 and 11.12±2.19, insulin dose 36.85±15.9 and 40.72±13.79, risk score based on IDF-DAR guidelines 10.61±3.52 and 11.2±2.8 respectively.

These two groups were analyzed based on age, gender, duration and type of DM, control of DM, presence or absence of macro vascular disease and risk score. Significant difference was found based on age and gender. Fifty four (69.2%) in age group >50 years did not fast versus 24 (30.8%) in age group 16-50 years. Twenty-two (42.3%) of participants age >50 years fasted versus 30(57.7%) in age group 16-50 years who didn’t fast (P-value 0.031). Based on gender in fasting group, there were 42(80.8%) female participants versus 10(19.2%) of male with significant P-value of 0.001.

**Table-III:** Complications related to hypoglycemia in studied population.

Parameters	Hypoglycemia Yes n (%)	Hypoglycemia No n (%)	Total n (%)	P-value
***Type of DM***				0.241
Type I DM	8(57.1)	6(42.9)	14(10.8)	
Type-2 DM	40(34.5)	76(65.5)	116(89.2)	
***Pregnancy***				0.238
Within target	2(4.2)	2(2.4)	4(3.1)	
Not within target	6(12.5)	2(2.4)	8(6.2)	
Not pregnant	40(83.3)	78(95.1)	118(90.8)	
***Type of Insulin***				0.873
Basal Bolus	20(41.7)	32(39.0)	52(40.0)	
Premix	26(54.2)	44(53.7)	70(53.8)	
Basal only	2(4.2)	6(7.3)	8(6.2)	

Overall, 48(36.9%) patients had at least one episode of hypoglycemia (<70mg/dl) in which 28(58.3%) patients were in the fasting group and 24(29.3%) in the non-fasting group. Overall, 58 (70.7%) patients in the non-fasting group did not experience any hypoglycemic episodes at all during Ramadan (P-value 0.021). Forty-two of these patients with hypoglycemia needed assistance. Hypoglycemia occurred more in patients who were on premix insulin 26(45%) than those on basal bolus 20(41.7%) and basal only 2(4.2%). Among twelve pregnant patients, eight experienced hypoglycemia, six of them were not with in target. Among fourteen patients with type-1 diabetes, eight of them had hypoglycemia. On the other hand, hyperglycemia (>300mg/dl) was observed in 72(55.4%) patients, significantly more in non-fasting group with 32(55.2%) patients versus only 20(27.8%) in fasting group (P-value 0.025). None of the participants had DKA/HONK.

## DISCUSSION

This study was conducted to apply new IDF DAR guidelines which provides an insight based on available data to classify patients on the basis of risk categories that were reclassified in the second addition in 2021. In this edition, the risk categorization was changed from informal categories to the one based on scoring system. This study was conducted based on this new scoring system which changed patients’ classification to fast or not to fast in many cases. In this study, only those patients having high risk score (> 6.0) as per new guidelines were enrolled.^10^ Among all participants, 78(60%) followed medical advice and did not fast and 52(40%) patients fasted during month of Ramadan despite advice against that. The DAR BAN study was conducted in Bangladesh, showed that 73.9 % patients intended to fast in high-risk group which were similarly advised against fasting.^11^

In this study, having high-risk patients based on IDF-DAR guidelines, 58.3% (n=28) participants, fasted during Ramadan had hypoglycemia, which is very high risk as compared to other studies. CREED was a landmark trial enrolling patients from multiple countries to describe patients approach to diabetes while fasting during Ramadan in 2010.^12^ The overall incidence of documented hypoglycemia during Ramadan was 7.1% and overall incidence of hypoglycemia in high risk was 9.2% and 13.8% in very high-risk participants.

In this study, 49.2% (n=64) patients were on insulin alone while 50.8% (n=66) were using both insulin and oral anti diabetic medications including metformin, sodium glucose linked transporter 2(SGLT2) inhibitors and dipeptidyl peptidase (DPP4) inhibitors. Hypoglycemia was more in patients using premix 54.2% (n=26) than on basal bolus 41.7%(n=20). In CREED trail, similar results were observed where patients on insulin regimens were having more risk for hypoglycemia as compared to those who were on oral antidiabetic medications. Those with type-2 diabetes on insulin 16.8% had a hypoglycemic episode. Those with type-2 diabetes and taking premix insulin, hypoglycemia occurred in 20.5% when compared with long-acting insulin 12.7%.

Another landmark study in Ramadan, the EPIDAR study conducted in 13 Muslim countries enrolling more than 12000 people showed that 43% of patients with type-1diabetes and 79% of patients with type-2 diabetes fasted during Ramadan.^13^ In our study with high-risk diabetic patients only based on IDF-DAR guidelines (risk score >6.5), 40% (n=52) patients fasted during Ramadan. This shows that near half of patients do fast taking all the associated risk even when advised against fasting in the studied population. This is tied with all the expected complications of hypoglycemia and hyperglycemia. EPIDAR showed that there is 4.7 times risk for type I diabetes and 7.5 times risk for type-2 diabetes of hypoglycemia in Ramadan. In this study which mainly comprise of type-2 diabetic patients, 58.3 % (n=28) patients had at least one hypoglycemia episode. EPIDAR participants had fivefold increase in hyperglycemia in patients with type-2 diabetes during Ramadan while 27.8% (n=20) patients in our study experienced hyperglycemia (>300mg/dl). There were fourteen participants with type-1 in our study in which eight participants had hypoglycemia.

The basic preparation for Ramadan starts before Ramadan which the most important preparatory period that can be continued in Ramadan and later. The pre-Ramadan education, adjustment of medications and risk stratification can decrease risk of developing complications in Ramadan. A systemic review which included seventeen studies with various study designs to evaluate significance of a Ramadan-focused diabetes education on hypoglycemic and other metabolic and clinical outcomes showed significant reduction in hypoglycemia risk in intervention groups who received education as compared with conventional group.^14^ The current study showed opposite trend to above in those not complying with physician advice not to fast and had more hypoglycemia.

Ramadan education program significance is evaluated by many others with almost similar results on biochemical parameters and the risk of hypoglycemia in patients with type-2 diabetes mellitus. Hypoglycemia being the main outcome due to significant morbidity and mortality is significantly reduced with better A1c control is an established fact. There has been a significant effect on lipids with reduction in low-density cholesterol (LDL) and improvement of high-density cholesterol (HDL).^15^ Yet another study done in UK, retrospectively analyzed two groups in which group A received all related education and group B didn’t get specific components of pre-Ramadan education. This study showed that there was significant weight reduction along reduction in risk of hypoglycemia.^16^

As our study has shown that despite being very high risk and advised against fasting significant number of participants fasted during Ramadan. This highlight importance of pre-Ramadan diabetes focused education to help these people prevent adverse events during Ramadan. Similarly, adjustment of anti-diabetic medications, insulin type and dosing greatly impact incidence of adverse events during Ramadan in people living with diabetes. A systemic review of insulin management recommendations in type-2 diabetic patients during Ramadan to improve glycemic control and reduce hypoglycemic events found that hypoglycemia post sahoor can be reduced by decreasing morning insulin dose while postprandial surge can be mitigated by increasing iftar dose of insulin contrary to normal days.^17^

Studies have also investigated the effect of using analogue insulin compared to human insulin. Human insulin 70/30 use in patients with Type-2 diabetes in Ramadan when compared with insulin lispro Mix25 resulted in better glucose control before and after Iftar with less glycemic variations.^18^ The long acting basal analogue insulin works better by having flat curve and less chances of hypoglycemia for obvious reasons. Glargine has been studied to be safe and effective and results in less glycemic variations alone or in combination with other medications.^19^ Sulphonylurea being secretogogue is more likely linked to hypoglycemia especially in Ramadan. The guidelines advocate its use in Iftar and newer short acting molecules to mitigate its hypoglycemia risk in Ramadan.^20^

The present study has provided an overview of the trend to follow medical advice during Ramadan and complication developed in high-risk diabetic patients. Health care professional should apply these guidelines and risk stratify patients. Those in high risk need to be followed thoroughly and much emphasis need to be put on risk management and breaking the fast in case of impending hypo or hyperglycemia for safe fasting. This study will pave way for further large-scale studies in local diabetic population. However, large scaler studies are needed to look for the other risk categories as well.

### Limitations:

This was a single center study and a multicenter study would yield more precise results.

## CONCLUSION

Patients with both type-1 and type-2 diabetes who fast during Ramadan represent a challenge to their physicians as almost half of patients (40%) did not follow medical advice and significant number of patients had hypoglycemia. There is need of more intense education before fasting, need to involve religious scholars and to disseminate guidelines based on local practices, and to conduct large scale studies to assess the impact of fasting in patients with diabetes.

### Author’s Contribution:

**QF:** Conceived, designed, reviewed literature, performed statistical analysis & drafted manuscript.

**TG:** Participated in data collection, literature review and interpretation of data, and helped in draft.

**SEM:** Helped in analysis and interpretation of data, and critically revised the manuscript.

**AHA:** Conceived, designed, and critically revised the manuscript.

All authors provided final approval for publication of the manuscript and are responsible for the integrity of the study.
